# Epidemiology of Surgical Site Infections Considering the NHSN Standardized Infection Ratio in Hip and Knee Arthroplasties

**DOI:** 10.3390/ijerph17093167

**Published:** 2020-05-02

**Authors:** Róża Słowik, Małgorzata Kołpa, Marta Wałaszek, Anna Różańska, Barbara Jagiencarz-Starzec, Witold Zieńczuk, Łukasz Kawik, Zdzisław Wolak, Jadwiga Wójkowska-Mach

**Affiliations:** 1 St. Luke’s Provincial Hospital, 33-100 Tarnów, Poland; rozaslowik@interia.eu (R.S.); malgorzatakolpa@interia.pl (M.K.); mz.walaszek@gmail.com (M.W.); arwena332@wp.pl (B.J.-S.); witoldz63@vp.pl (W.Z.); l.kawik@wp.pl (Ł.K.); zdzich_w@interia.pl (Z.W.); 2 State Higher Vocational School, 33-100 Tarnów, Poland; 3 Department of Microbiology, Jagiellonian University, Polish Society of Hospital Infections, 31-007 Kraków, Poland; mbmach@cyf-kr.edu.pl

**Keywords:** surgical site infection, hip arthroplasties, knee arthroplasties

## Abstract

Introduction Surgical site infections (SSIs) are a predominant form of hospital-acquired infections in surgical wards. The objective of the study was analysis of the incidence of SSI in, both primary and revision, hip and knee arthroplasties. Material and methods: The study was conducted in 2012–2018 in a Trauma and Orthopedics Ward in Tarnów according to the methodology of the Healthcare-Associated Infections Surveillance Network (HAI-Net), European Centre for Disease Prevention and Control (ECDC). Results: The surveillance comprised 2340 surgery patients, including: 1756 Hip Arthroplasties (HPRO) and 584 Knee Arthroplasties (KPRO). In the group of patients under study, 37 cases of SSI were detected, including: 26 cases of SSI after HPRO and 11 cases in KPRO. The average incidence of SSI amounted to 1.6% (1.5% HPRO and 1.9% KPRO) and in-hospital incidence density rates were 1.23 and 1.53 per 1000 patient-days, respectively. Median age of surgical patients in both HPRO and KPRO was 70 years. Women were undergoing arthroplasty surgery more often than men, HPRO (*p* < 0.05) and KPRO (*p* < 0.001). Patients with SSI stayed in the ward longer (SSI-HPRO, *p* < 0.001) (SSI-KPRO *p* < 0.01). In KPRO operations, the incidence of SSI was higher than expected, calculated according to the Standardized Infection Ratio (SIR). The most common etiologic agents isolated from SSIs in both HPRO and KPRO were coagulase-negative staphylococci. Conclusions: Establishing a thorough surveillance of hospital-acquired infections that takes into consideration epidemiological indicators is indispensable to properly assess the epidemiological situation in the ward. The optimal solution is to carry out long-term and multi-center surveillance in the framework of a uniform program, however, even results of single-center studies provide valuable data indicating challenges and needs in improving patient safety.

## 1. Introduction

Healthcare-associated infections (HAIs) are present in all areas of medicine, however, in surgical wards, surgical site infections (SSIs) are most prominent as regards this type of infections [1]. One of the factors determining the development of SSI in the trauma and orthopedic wards is the specific nature of this field of medicine associated with procedures involving bone tissue, which is especially prone to infection [2,3]. Surgical site infections are the cause of prolonged hospitalization and increased costs of treatment, they lower the patient’s quality of life, may delay the patient’s return to work, cause disability or even lead to death [4,5]. The incidence of SSI depends on many factors, such as: the patient’s preparation for surgery or the course of the perioperative and postoperative periods [6,7,8]. Mangram et al. [9], in their handbook concerning the prevention of SSI, list the risk factors for infection that are patient- and surgery-dependent. In the literature, incidence rates of SSI in the trauma and orthopedic wards vary greatly. In the 2008–2009 ECDC studies, the incidence of SSI in HPRO amounted to 1.2% and in KPRO it was 0.8% [10]. Poland did not participate in the aforementioned European survey, therefore the incidence rates of SSI in Poland as regards HPRO and KPRO are known only from single-center studies. In a single-center study by Wójkowska-Mach et al., carried out in Kraków, Poland, in 2008, the incidence of SSI in HPRO was 2.3% and in KPRO 7.0% [11]. In a subsequent study conducted by Pawłowska et al., in Sosnowiec, Poland, the incidence of SSI in HPRO amounted to 5.8% and in KPRO 5.4% [12].

Drawing conclusions from studies which are not based on representative numbers of settings should be very careful. However, there are probably some areas that need improvement as regards prevention and control of infections in this kind of procedures. According to the latest ECDC protocol (from 2017) for surveillance of SSIs, specific process and outcome measures are needed in order to value comparison of the epidemiological data [13]. Among these data, there are:

- the amount of alcohol hand rub used,
- the presence of a system for root cause analysis/review of SSIs,
- the rate of proper antibiotic perioperative prophylaxis,
- the mode of preoperative skin preparation including hair removal recommendations and type of surgical site skin disinfection.

According to this protocol, data on ensuring the patient’s normothermia in the perioperative period (within one hour of the end of operation) and intensive perioperative blood glucose control and blood glucose level monitoring for adult patients undergoing surgical procedures should also be taken into account for benchmarking the results of surveillance. Additionally, information that can prove helpful for the description of health-service quality may be the number of operating room door openings. Furthermore, it can be the type of post-discharge surveillance of SSI, especially in procedures with artificial item implantation. Unfortunately, so far these data were not routinely collected in the surveillance of SSI in Polish hospitals (this protocol has been used in European countries since 2018), and even patient-based surveillance was rare. The patient-based surveillance enables analysis of SSI considering the variables included in the Standardized Infection Ratio (SIR), and consequently a precise assessment of the occurrence of SSI, including the most important risk factors. However, such analyses for Poland have so far only been conducted in single-center studies. According to the latest ECDC protocol for the surveillance of SSI, mentioned here, also mortality connected with infections is an important indicator of orthopedic surgery quality. Though low, mortality after hip arthroplasty is an indicator that should be evaluated because of the large number of operations performed and the importance of predictive factors for outcome [14,15]. Unfortunately, there is a problem of obtaining these data in Poland, and this is also the indicator included only in the last version of the ECDC protocol [13].

The aim of this study is analysis of the epidemiology of SSIs after knee and hip arthroplasties, considering the factors included in the National Healthcare Safety Network (NHSN) risk index.

## 2. Material and Methods

2340 patients were examined in the Trauma and Orthopedic Ward of St. Luke’s Provincial Hospital in Tarnów, Poland, in 2012–2018. In compliance with The International Classification of Diseases, Ninth Revision, Clinical Modification (ICD-9-CM), and with the methodology of the National Healthcare Safety Network (NHSN), the operations performed were divided into: hip arthroplasties (HPRO, ICD-9 00.70–00.73; 00.85–00.87; 81.51–81.53) and knee arthroplasties (KPRO, ICD-9: 00.80–00.84; 81.54; 81.55), both primary and revision, taken together-as in NHSH. No of patient days was 20609 for HPRO and 7177 for KPRO.

Active surveillance of SSI infections in the ward under study was commenced in 2008, and the results of this surveillance have already been published [16,17]. Data on patients were collected by nurse epidemiologists through active monitoring of hospital-acquired infections, in which the results obtained from microbiological and analytical tests and medical documentation along with the assessment of the patients’ clinical condition were analyzed daily. The methods for the identification of SSI employed in the study were consistent with the Healthcare-Associated Infections Surveillance Network (HAI-Net), European Centre for Disease Prevention and Control (ECDC) [18,19,20]. The follow-up period lasted 90 days from the operation date, for deep and organ-space infections, and 30 days for superficial. The detected cases of SSI were consulted with the patient’s attending physician. Microbiological testing involved material consisting of swabs collected from the surgical site or intraoperative samples, and microbiological diagnostics were carried out in the Vitek 2 Compact automated system (bioMérieux, Inc. 100 Rodolphe Street Durham, NC, USA).

The analysis of data took into consideration the following indicators [21,22]: (a) incidence of SSI (b) incidence density, (c) SSI risk index according to the National Healthcare Safety Network (NHSN), formerly known as the National Nosocomial Infections Surveillance System (NNIS) and (d) Standardized Infection Ratio (SIR).

### 2.1. SSI Incidence and Incidence Density

Incidence is a measure of disease frequency. It determines the proportion of subjects who experienced SSI during a block of time, without taking into account when SSI developed in the subjects. It is calculated using the following formula: (Number of SSI × 100)/Number of operations [23]. The incidence density of in-hospital SSIs per 1000 post-operative patient-days included SSIs diagnosed during the hospital stay in patients with a known discharge date from the hospital [10].

### 2.2. The NHSN Risk Index and SIR

The CDC-NHSN risk index consists of three risk factors that affect SSI incidence, i.e.,: patient’s condition expressed by ASA score, surgical wound microbiological contamination and duration of the surgery. A detailed description of ASA categories and microbiological wound classes are provided in the ECDC protocol [13]. A score of 0-3 is assigned based on the sum of values derived from the American Society of Anesthesiologists physical status classification (ASA) score [13,24], the surgical wound class (SWC), marked in Table 1 as W [21], and operation duration in relation to a procedure-specific length marked in Table 1 as T. In the NHSN risk index, one point is assigned when the wound is “contaminated” or “dirty”, another point if the duration of surgery exceeds the 75th percentile of the expected duration for a given type of operation, and one point if the patient has an ASA score higher than 2. Therefore, for each procedure, the NHSN risk index may range from 0 to 3 [9,19]. The Standardized Infection Ratio (SIR) compares the actual number of healthcare associated infections (HAIs) at each hospital to the predicted number of infections. The formula for calculating the SIR is: SIR = actual number of infections/predicted number of infections [22]. SIR > 1.0 indicates that there are more SSIs than predicted, and conversely, SIR < 1.0 indicates that there are fewer SSIs than predicted.

Statistical analysis of the collected material employed the IBM SPSS software, SPSS-*Statistical Package for the Social Sciences* (STATISTICS 24, Armonk, NY, USA), and Microsoft Excel, Microsoft Office 2016, (Redmond, WA, USA). Statistical indicators that were applied in the study: arithmetic mean, median, standard deviation, 95% confidence interval, and for features of ordinal or dichotomous character, information on the number and percentage share was used. To compare the frequency of occurrence of qualitative feature variants, Pearson’s chi-square test of independence was employed. Fisher’s exact test was used for variables of small numbers and the ANOVA test for quantitative variables.

The use of data was approved by the BioethicalCommittee of the Jagiellonian University (No. KBET/122.6120.118.2016 from May 25, 2016).

## 3. Results

During the study, 1756 HPRO surgeries were carried out, in which the median age of patients was 70 years, for women 73 years, and for men 67 years. KPRO operations were performed on 584 patients. The median age of patients was 70 years, for women 70 years, for men 69 years (Table 2). 26 cases of SSI were detected in HPRO surgeries (incidence of 1.5%), and 11 cases of SSI were revealed in KPRO operations (1.9%) (Table 3). The in-hospital incidence density of SSI was 1.26 HPRO and 1.53 KPRO.

During the study, the median duration of patients’ stay in the ward for both HPRO and KPRO was 10 days and the median waiting time for the procedure was 2 days. Median time of HPRO procedure was 85 min and of KPRO-105 min. Patients undergoing HPRO surgery who demonstrated the presence of SSI stayed in the ward longer than patients without infection, 17 days vs 10 days (*p* < 0.001). It was similar in the case of KPRO surgeries as the median number of days of stay for a patient with SSI was 22 vs 9 days for a patient without infection (*p* < 0.01) (Table 3).

The incidence of SSI in HPRO, considering risk factors, amounted to: 0.6% for patients without risk factors; 1.9% for patients with one risk factor; 1.2% for patients with two and three risk factors (Table 4). The Standardized Infection Ratio did not exceed 1 and the average incidence of SSI after HPRO was at a comparable level to the average incidence in European countries.

The SSI incidence in KPRO, considering risk factors, amounted to: 3.0% for patients without risk factors; 1.8% for patients with one risk factor; 8.3% for patients with two and three risk factors (Table 4). A high risk of developing SSI infection in KPRO operations was demonstrated. The Standardized Infection Ratio exceeded 1 at all three levels of observation and amounted to: 7.0 for patients without risk factors, 2.0 for patients with one risk factor and 2.0 for patients with two and three risk factors (Table 4).

The etiologic agents for both HPRO and KPRO were predominantly coagulase-negative staphylococci (CoNS), which accounted for 35.1%, and *Staphylococcus aureus*, which was responsible for 18.9% of SSIs. All strains of *S. aureus* were susceptible to methicillin, vancomycin and linezolid. The rods Enterobacteriales with extended-spectrum beta-lactamase (ESBL) resistance mechanism accounted for 1 (8.3%). As regards non-fermenting rods, *Acinetobacter baumannii* 1 (100%) was completely resistant to cefepime, cefotaxime, ceftazidime and ciprofloxacin but retained carbapenem sensitivity (Table 5).

Among the CoNS strains, 12 (92.3%) were MR CoNS strains; all strains of S. aureus were susceptible to methicillin, vancomycin and linezolid; ESBL Enterobacteriales rods accounted for 1 (8.3%); Acinetobacter baumannii 1 (100%) was completely resistant to cefepime, cefotaxime, ceftazidime and ciprofloxacin with retained sensitivity to carbapenems.

## 4. Discussion

In the ward under study, HPRO surgeries were among the most common. The results of European research also indicate that this type of operation (HPRO) is frequently performed in Europe [25]. In ECDC research, in 2008–2009, the incidence of SSI in HPRO amounted to 1.2% [10], and the incidence taking into account risk factors was as follows: 1.4% (0 risk factors); 2.4% (1 risk factor); 3.3% (2 and 3 risk factors) [10]. In American NHSN studies, carried out in 2006–2008 [26], the average incidence of SSI was lower than the one in Europe and amounted to: 0.7% (0 risk factors); 1.4% (1 risk factor); 2.4% (2 and 3 risk factors). The mean cumulative incidence rate of SSI after HPRO reported in European countries in 2017 [27] was 1.0%, so the proportion observed in our study, that is 1.5%, is higher than in American NHSN and European ECDC projects. For the in-hospital incidence density rates, the rate in our study was 1.23 per 1000 patient days in hospital, compared to 0.3 in the ECDC project [27].

Unfortunately, the results for KPRO are worse as the ward studied by us revealed a high incidence of 1.9% associated with SSI following KPRO operations. Thus, it was 2 times higher than the data from HAI-Net for 2008–2009 [10], 3 × higher than the data from HAI-Net for 2014 [25], in which the average European incidence was 0.6%, and almost 4 times higher when comparing to the mean rate value for 2017—0.5%. The in-hospital density rate in our study was also higher than in the ECDC report since the values obtained were 1.45 and 0.1, respectively [27].

The present data are particularly noteworthy as regards significant differences concerning in-hospital incidence density rates, in Poland four times higher for HPRO and 14.5 times for KPRO! However, in the analyzed hospital, a significant postoperative hospital stay was simultaneously uncovered, about 2 times longer than the medians reported in the ECDC data, i.e., 10 days in Poland vs. 6 days in EU countries (HPRO), and 11 days vs. 5 days KPRO [27]. So maybe, in Poland, more SSIs are qualified during the first hospitalization. But, this unfortunately does not explain such a significant difference concerning in-hospital incidence density of SSI. It is especially so, as Poland probably exhibits low sensitivity of post-discharge surveillance of SSI. This problem was reported in the studies of SSI following thoracic surgery and after cesarean sections [28,29].

On the other hand, the data presented also point to an alarmingly long hospitalization, as confirmed by Schlosser et al. who stated that in the USA the median length of stay in 2016–2019 was 2.0 days-the data came from a large healthcare system that includes over 180 affiliated medical centers across the United States and involved 1,824 hip and knee joint arthroplasties [30].

For a comprehensive assessment of the results obtained, and thus for the effectiveness of the infection surveillance program, data on structure and process indicators would undoubtedly be helpful. However, they were not included in the study protocol, and at the European level they are also still in the process of implementation, acquisition and analysis. Among the process indicators describing the quality of care, there is also the number of operating room door openings. It is associated with a risk of air contamination and its uncontrolled movement in the operating room, which in the case of clean field operations associated with implant placement may play a special role. Agodi et al., in a study conducted in 2011 in 28 operating rooms, with different ventilation systems, for total joint replacement surgery, showed that the number of door openings and the number of people correlate with the index of microbial air contamination [31]. Breier et al., in a study covering 33,463 HPRO and 7749 KPRO in 48 German hospitals in the period from 2004 to 2009, checked the effect of laminar airflow ceiling size on infection rates following hip and knee prostheses [32]. These authors showed that neither LAF nor the size of the LAF ceiling were associated with lower infection risk, however, incidence rates reported in their studies were low, for elective hip and knee prostheses they were, respectively, 0.74 and 0.63 per 100 procedures.

In our study, apart from not fully satisfactory incidence rates, also the analyses with the use of SIR: patient stratification and analysis of incidence according to the risk index yielded disturbing and unexpected results, although without any statistical significance (for HPRO *p* = 0.419 and KPRO = 0.64). In the population under study, quite high incidence rates were obtained for the patients with one risk factor or without risk factors. There was also a lack of statistical significance in the case of incidence among patients with many risk factors compared to other groups. These results indicate that the problem of excessive risk of SSI development concerns healthy patients without risk factors undergoing primary prosthesis surgery (clean microbiological site) while the surgery is not excessively prolonged. In this group of patients “without risk factors”, the development of SSI may be affected by the quality of patient surveillance in the course of preparation for surgery and during the procedure. Perhaps the initial good condition of the patient triggers routine activities and does not trigger the need to reflect on due diligence in assessing other risk factors of SSI, such as: age, body weight, nutritional status, stimulants, and others. These factors were not the subject of this analysis, however, they were deemed significant by Mangram et al. [9]. The justification for this hypothesis may be the bureaucracy model known in sociology which highlights dysfunctions including the lack of efficient operation in unusual situations that are not covered by regulations (procedures). On the other hand, these results indicate that in the case of registration of infections characterized by low incidence rates, it is necessary to conduct long-term observation, and optimally observation in many centers in a given country or region, to obtain accurate data, also necessary for statistical analyses. Analysis of demographic data, limited in extent only to age in relation to the studied population, demonstrates that Polish patients undergoing implant orthopedic surgery are younger than their European counterparts.

Moreover, some elements connected with the organization and methods of treatment are probably different in Polish hospitals, which may be evidenced by a comparison of the durations of surgery. In ECDC research from 2008–2009 [10], the median duration of HPRO surgery for European countries was 76 min, on average, which was very close to the median duration obtained in our study (80 min). However, surprisingly, a few reports from Poland talk about durations of procedures that are significantly longer. Data from 2005 indicate 120 min [4], and from 2013–2015 (median) even 140 min [12]. The authors of these studies also point to significant problems associated with high incidence of SSI in HPRO and KPRO, several times exceeding the incidence in the tested center, as the SSI incidence rate was 5.8% vs. 4.4%, which may confirm the hypothesis that the duration of surgery significantly affects the incidence rates. The European studies carried out as part of the European program of surveillance of KPRO SSI in 2008–2009 cite the median duration of surgery as 80 min [10] and in a later study conducted in 2012–2013 [33] it was 79 min. In the ward studied by us, the median KPRO surgery duration was 105 min. While in a study conducted by Pawłowska et al. [11], the median KPRO surgery duration amounted to 110 min. Hence, the duration of KPRO operations was longer in Polish studies than in Europe.

When considering HPRO and KPRO operations in the studied ward, the most common pathogens responsible for SSIs were coagulase-negative staphylococci (35.1%) and *Staphylococcus aureus* (18.9%), as well as others. Malhas et al. [34] in their study concerning SSI-D, obtained comparable results as regards etiologic agents in HPRO and KPRO, i.e., percentages of SA and CoNS at the levels of 36% and 35%, respectively. In previous analyses by other authors from 2008–2012, the dominance of *Staphylococcus aureus* (40%) was observed in all forms of SSI, which was in line with the European trend, in which in a HAI-Net study from 2013–2014, *Staphylococcus aureus* concerned 30.8% and CoNS 18.8% of SSI cases [22]. In our study, 92.3% of CoNS strains cultured from deep SSI were methicillin resistant (MR). In the research by Malhas et al. [34], concerning SSI-D (HPRO and KPRO revisions), CoNS resistance to methicillin increased during the period of the study from 63% to 70%, respectively, and similar to our study, was higher than in the case of SA. This situation may affect future antibiotic prophylaxis regimens.

### Limitation and Strengths of the Study

The basic limitation of our study is the fact that this is a single-center study. The others are: lack of validation of the surveillance (and also the control of compliance with specific procedures of SSI prevention), lack of a systemic solution for post-discharge surveillance and lack of data on structure and process measures included in the protocol. However, in our opinion, the overwhelming strength is the surveillance with the use of a patient-based protocol, extremely rare in Poland, which allowed us to obtain data stratified by main risk factors (NHSN risk index and SIR) and carry out analyses based on data gathered in the period of seven years.

## 5. Conclusions

HPRO and KPRO operations more often involved women and the patients operated were younger in comparison to European studies. Surgical site infections resulted in extending the duration of hospitalization by 7 days for hip arthroplasties and by 13 days for knee arthroplasties. The SSI incidence in HPRO remained at a level not markedly higher than average incidence in other European countries, but it was significantly higher in KPRO operations, particularly in the group of patients without the studied risk factors (surgical site cleanliness, surgery duration and general condition of the patient expressed by the ASA score). Additionally, in-hospital incidence density for both HPRO and KPRO were much higher in our study than the ones reported by ECDC. Establishing a thorough surveillance of hospital-acquired infections that takes into consideration epidemiological indicators is indispensable to properly assess the epidemiological situation in the ward and as a result to improve quality of care and patients safety. The results of single-center studies may be a factor broadening the knowledge in the field of hospital infections surveillance both in the examined ward and at the country level in Poland. This publication can help to promote preventive measures based on scientific evidence. It can also become an incentive for continuous improvement of the system of surveillance through systematic collection and analysis of data on hospital-acquired infections.

## Figures and Tables

**Table 1:** Studied risk factors of SSI.

Risk Factor	Point = 0, When:	Point = 1, When:
Surgical site cleanliness	W1, W2	W3, W4
ASA * score classification	ASA 1, ASA 2	ASA 3, ASA 4, ASA 5,
Surgery duration (T) above the 75th percentile	≤T	>T
SSI risk index = **	Total points	

* ASA-the American Society of Anesthesiologists physical status classification ** The CDC-NHSN risk index consisting of three risk factors that affect SSI incidence.

**Table 2:** Patient age on the day of surgery, considering the type of operation, gender and median in 2012–2018.

Patient Age on the Day of Surgery [Years]		
Number of operations	HPRO	KPRO
	1756	584
Patient age [years]		
Mean (95% Cl) *	70 (69–70)	69 (68–70)
Standard deviation	11.9	8.3
Median	70	70
Age of women on the day of surgery [years]		
Mean (95% Cl) *	71 (71–73)	69 (68–70)
Standard deviation	11.4	8.1
Median	73	70
Age of men on the day of surgery [years]		
Mean (95% Cl) *	66 (66–68)	68 (67–70)
Standard deviation	11.9	9.0
Median	67	69
Patient gender [*n* (%)]		
Woman	1038 (59.1)	450 (77.1)
Man	718 (40.9)	134 (22.9)
Total	1756 (100)	584 (100)
Pearson’s chi-square	*p* < 0.05	*p* < 0.001

* (95% CI)-95% confidence interval for the mean.

**Table 3:** Number of days of patients’ stay in the ward, waiting time for surgery, operation duration, number of days of patients’ stay in the ward by type of surgery and patients with or without SSI.

Procedure Description	HPRO	KPRO
Number of operations	1756	584
All SSIs	26	11
Incidence of SSI per 100 operations	1.5	1.9
In-hospital incidence density per 1000 patient days	1.26	1.53
Length of stay in the ward [days]		
Mean (95% Cl) *	12 (11–12)	13 (12–13)
Standard deviation	7.200	7.800
Median	10	10
Waiting time for surgery in the ward [days]		
Mean (95% Cl) *	2 (3–3)	2, (2–3)
Standard deviation	5.034	3.580
Median	2	2
Surgery duration [minutes]		
Mean	88 (86–91)	102 (98–106)
Standard deviation	37.0	37.9
Median	85	105
Length of stay in days for patients with SSI and without SSI in the Trauma and Orthopedic Ward		
	HPRO	KPRO
ANOVA test (*p*)	<0.001	<0.01
Patients with SSI (days)	17	22
Patients without SSI (days)	10	9


^*^(95% CI)-95% confidence interval for the mean.

**Table 4:** Incidence of SSI in 2012–2018 in patients of the Trauma and Orthopedic Ward compared with NHSN (2006–2008) including risk factors.

		The Studied Ward					
Surgery type	Risk factors	HPROs [no]	SSIs [no]	Incidence (%)	Incidence in EU countries [9] (%)	Expected no of SSIs	SIR * SSI
HPRO *p* = 0.419	No risk factors	523	5	0.6	1.4	7	0.7
	1 risk factor	467	9	1.9	2.4	11	0.8
	2 and 3 risk factors	88	1	1.2	3.3	3	0.3
KPRO *p* = 0.264	No risk factors	230	7	3	0.6	1	7
	1 risk factor	113	2	1.8	0.9	1	2
	2 and 3 risk factors	24	2	8.3	1.6	1	2

SIR * SSI-SSI Standardized Infection Ratio. The expected number of SSIs was calculated according to the formula = SSI cumulative incidence in the ECDC program [10] × number of (own) operations performed/100. SIR for SSI was calculated according to the formula = number of SSIs detected/number of expected SSIs. SSI-surgical site infection, *p*-Pearson’s chi square.

**Table 5:** Microorganisms isolated from patients with SSI in 2012–2018.

Microorganism	HPRO	KPRO	Total
	*n* (%)	*n* (%)	*n* (%)
Coagulase-negative staphylococci (CoNS)	9 (34.6)	4 (36.4)	13 (35.1)
*Staphylococcus aureus*	4 (15.4)	3 (27.3)	7 (18.9)
None isolated	4 (15.4)	1 (9.1)	5 (13.5)
*Klebsiella pneumoniae*	3 (11.5)	1 (9.1)	4 (10.8)
*Acinetobacter baumannii*	1 (3.8)	2 (18.2)	3 (8.1)
*Escherichia coli*	1 (3.8)		1 (2.7)
*Proteus mirabilis*	1 (3.8)		1 (2.7)
*Citrobacter freundii*	1 (3.8)		1 (2.7)
*Enterococcus faecium*	1 (3.8)		1 (2.7)
*Serratia liquefaciens*	1 (3.8)		1 (2.7)
Total	26 (100.0)	11 (100.0)	37 (100.0)
